# Evaluation of T-Cell Responses Against Shared Melanoma Associated Antigens and Predicted Neoantigens in Cutaneous Melanoma Patients Treated With the CSF-470 Allogeneic Cell Vaccine Plus BCG and GM-CSF

**DOI:** 10.3389/fimmu.2020.01147

**Published:** 2020-06-05

**Authors:** Enrique Podaza, Ibel Carri, Mariana Aris, Erika von Euw, Alicia Inés Bravo, Paula Blanco, Juan Manuel Ortiz Wilczyñski, Daniel Koile, Patricio Yankilevich, Morten Nielsen, José Mordoh, María Marcela Barrio

**Affiliations:** ^1^Centro de Investigaciones Oncológicas, Fundación Cáncer, Buenos Aires, Argentina; ^2^IIBIO, UNSAM, San Martín, Buenos Aires, Argentina; ^3^UCLA JCCC-Translational Oncology Research Labs, Los Angeles, CA, United States; ^4^T-Cure Bioscience Inc., Los Angeles, CA, United States; ^5^Centro de Excelencia en Medicina Translacional, Hospital El Cruce, Buenos Aires, Argentina; ^6^Laboratorio de Trombosis Experimental- IMEX-ANM, Buenos Aires, Argentina; ^7^Plataforma Bioinformática, INBioBA-MPSP, Buenos Aires, Argentina; ^8^Department of Health Technology, The Technical University of Denmark, Lyngby, Denmark; ^9^IIBBA-CONICET, Fundación Instituto Leloir, Buenos Aires, Argentina; ^10^Instituto Alexander Fleming, Buenos Aires, Argentina

**Keywords:** cutaneous melanoma, CSF-470 allogeneic cell vaccine, neoantigens, tumor associated antigens, melanoma immunotherapy

## Abstract

The CSF-470 vaccine consists of lethally-irradiated allogeneic cells derived from four cutaneous melanoma cell lines administered plus BCG and GM-CSF as adjuvants. In an adjuvant phase II study vs. IFN-α2b, the vaccine significantly prolonged the distant metastasis-free survival (DMFS) of stages IIB-IIC-III melanoma patients with evidence of the induction of immune responses against vaccine cells.

**Purpose:** The aim of this study was to analyze the antigens against which the immune response was induced, as well as the T-helper profile and lytic ability of immune cells after CSF-470 treatment.

**Methods:** HLA-restricted peptides from tumor-associated antigens (TAAs) were selected from TANTIGEN database for 13 evaluable vaccinated patients. In addition, for patient #006 (pt#006), tumor somatic variants were identified by NGS and candidate neoAgs were selected by predicted HLA binding affinity and similarity between wild type (wt) and mutant peptides. The patient‘s PBMC reactivity against selected peptides was detected by IFNγ-ELISPOT. T-helper transcriptional profile was determined by quantifying GATA-3, T-bet, and FOXP3 mRNA by RT-PCR, and intracellular cytokines were analyzed by flow cytometry. Autologous tumor cell lysis by PBMC was assessed in an *in vitro* calcein release assay.

**Results:** Vaccinated patient‘s PBMC reactivity against selected TAAs derived peptides showed a progressive increase in the number of IFNγ-producing cells throughout the 2-yr vaccination protocol. ELISPOT response correlated with delayed type hypersensitivity (DTH) reaction to CSF-470 vaccine cells. Early upregulation of GATA-3 and Foxp3 mRNA, as well as an increase in CD4+IL4+cells, was associated with a low DMFS. Also, IFNγ response against 9/73 predicted neoAgs was evidenced in the case of pt#006; 7/9 emerged after vaccination. We verified in pt# 006 that post-vaccination PBMC boosted *in vitro* with the vaccine lysate were able to lyse autologous tumor cells.

**Conclusions:** A progressive increase in the immune response against TAAs expressed in the vaccine and in the patient's tumor was induced by CSF-470 vaccination. In pt#006, we demonstrated immune recognition of patient's specific neoAgs, which emerged after vaccination. These results suggest that an initial response against shared TAAs could further stimulate an immune response against autologous tumor neoAgs.

## Introduction

Immunotherapy is changing the prognosis of some cancer patients through the use of monoclonal antibodies that block immune checkpoints (immune checkpoint blockade, ICKB), such as cytotoxic T lymphocyte antigen-4 (CTLA-4/CD28) and/or programmed cell death 1 (PD-1)/PD-1 ligand (PD-L1) axes. Release from suppressive checkpoints essentially attempts to force the immune system to attack tumor cells allowing their elimination. This is evidenced by the clinical responses obtained in advanced cutaneous melanoma (CM) and non-small cell lung carcinoma patients treated with ICKB (1, 2). However, clinical responses are only seen in about 30% of the patients, although a recent report has shown a strong impact of combined ICKB therapy on metastatic CM patient's overall survival (3). Also, ICKB has been recently shown to be effective as adjuvant treatment in stage III CM patients (4, 5). Blocking of regulatory axes may release lymphocytes from exhaustion, and achieve clinical effectiveness provided anti-tumor reactive lymphocytes already exist in patients in numbers high enough to achieve tumor control. Indeed, better results of ICKB have been reported in patients whose tumors were heavily infiltrated by lymphocytes before immunotherapy (6). Under this scenario, active immunization with vaccines can be revisited as an interesting treatment since it may favor tumor-reactive lymphocytes generation/augmentation before ICKB administration, thus enhancing the chances to induce destruction of tumor cells. The interest in cancer vaccination has emerged from several clinical studies demonstrating that vaccines could improve clinical outcome of immunotherapy protocols. Among them, we can mention the use of a granulocyte-macrophage colony-stimulating factor (GM-CSF) secreting tumor vaccine in combination with CTLA-4 blockade for metastatic prostate cancer (7) and the use of an autologous vaccine plus Bacillus Calmette–Guerin (BCG) which showed an increased response rate with subsequent ipilimumab for progressive disease in stage III melanoma pts (8). Our group has developed the therapeutic vaccine CSF-470, an allogeneic mixture of four lethally-irradiated CM cell lines co-adjuvated with BCG and human recombinant GM-CSF, for the adjuvant treatment of stages IIB–IIC–III CM patients. In a randomized phase II study, CSF-470 vaccine has demonstrated a significant benefit in distant metastasis-free survival (DMFS) vs. IFNα2b, with good tolerability and evidence of induction of adaptive and innate immune responses (9–11). RNASeq of CSF-470 has revealed that the vaccine is a source of multiple shared tumor associated antigens (TAAs), including melanoma differentiation antigens (MD), cancer testis (CT) and others (9) to stimulate the patient's immune system. The addition of BCG to each vaccination produces local inflammation and polarizes the immune system toward a Th1 response, also activating NK cells cytotoxicity and memory-like response (9, 12–14). Finally, injection of GM-CSF at low doses attracts monocytes to the vaccination site (12), which may differentiate into dendritic cells (DCs). This combination should favor antigen (Ag) uptake by macrophages and DC and boost adaptive immunity through cross-presentation to naïve lymphocytes, either locally or after migration to draining lymph nodes (14, 15). A previous vaccine (Canvaxin) tested in a Phase III clinical trial using irradiated allogeneic tumor cells plus BCG vs. placebo with BCG was interrupted due to lack of efficacy. However, in that study, allogeneic cells were not pretreated with cytokines, BCG was only injected twice, and no GM-CSF was used (16).

We have shown that repeated CSF-470 vaccinations stimulated a long-term cellular and humoral immunity response directed against vaccine Ags, and in two patients, a similar immune response was generated against autologous tumor Ags (12). Here, we investigated in depth the nature of the cellular immune response induced by vaccination. To that aim, we evaluated HLA-restricted immune responses to non-mutated TAA- derived synthetic peptides by PBMC obtained before and during the CASVAC-0401 protocol. In one patient, we could also analyze the immune response to tumor neoantigens (neoAgs) and autologous tumor cell lysis. CD4+ T cells profiles of 13 vaccinated patients were also analyzed. Our results suggest that CSF-470 induces an antitumor immune response targeting non-mutated TAAs as well as private neoAgs, which may prevent or delay melanoma relapse.

## Methods

### CASVAC-0401 Study and PBMC Samples From Vaccinated Patients

CASVAC-0401 study was a phase II clinical study (clinicaltrials.gov, NCT01729663) that compared CSF-470 plus BCG and recombinant human GM-CSF vaccination with medium dose IFN-α2b as 2 years adjuvant treatments in post-surgery CM patients stages IIB, IIC, and III. The CASVAC-0401 study was carried out after approval by the Ethics Committee of the Instituto Alexander Fleming, with written informed consent from all subjects in agreement with the Declaration of Helsinki. The informed consent included the authorization to publish the results obtained, providing anonymity was assured. The “Ethics Committee of the Instituto Alexander Fleming (Buenos Aires, Argentina)” is approved by the Central Ethics Committee of the City of Buenos Aires (Argentina). The study was also approved by the Argentine Regulatory Agency (ANMAT) (Disposition 1299/09).

Vaccinated patients received 13 vaccinations: the first 4 vaccines, 3 weeks apart; the next 5 vaccines, 2 months apart; and the last 4 vaccines, 3 months apart. During CASVAC-0401 protocol, PBMC samples were obtained at baseline (PRE), 6 (P1), 12 (P2), and 25 months (P3) after protocol start and frozen under liquid nitrogen (Supplementary Figure 1). A DTH score was determined in each vaccination. On the vaccination day, DTH was performed in the forearm with 1/10th of the CSF-470 dose. The reaction was measured at 1, 24, 48, and 72 h and recorded as follows: 0: macular erythema <0.5 cm diameter; 1: macular erythema 0.5–1.0 cm; 2: macular erythema 1.1–2.0 cm; 3: macular erythema > 2.0 cm; and 4: papular erythema > 2.0 cm. A DTH score corresponding to the sum of the four values was calculated for each vaccination. Details of the vaccination protocol and sample processing were published elsewhere (9, 12).

### HLA Typing

HLA haplotypes (class I and class II) were determined on PBMC isolated from 13 vaccinated patients by Scisco Genetics (17) (Supplementary File 1, ST1–ST2).

### HLA-Restricted Non-mutated TAAs Peptides Selection for ELISPOT Testing

For each evaluated patient (*n* = 13), we selected HLA-class I and HLA-class II restricted peptides corresponding to non-mutated TAAs frequently expressed in CM, which were expressed in the vaccine cells. Peptides were selected mainly from the TANTIGEN DataBase (http://projects.met-hilab.org/tadb/) and a few of them from the literature. Selected peptides were either T cell epitopes previously identified in functional assays (*in vitro* and/or *in vivo*) or HLA ligands as determined by physical detection (18). For pt#006, three additional predicted peptides (strongest predicted binders to HLA-A^*^1101) from PRAME, RAB38/NY-MEL-1, and GRP143 TAAs, respectively, were included (Supplementary File 1, ST3 and ST4).

### Neoantigen Prediction

WES of frozen Pt#006 C-mts (tumor) paired with PBMC (germline) was performed after total DNA isolation, as previously described (10). To identify somatic single-nucleotide variants (SNVs) present in the C-mts, we used MuTect2 (19) from GATK version 3.8-0. Mutect2 was applied using tumor paired with germline WES data, Cosmic version 82 (20), dbSNP build 138 (21), removing soft clipped bases, and setting a TLOD threshold of 5.88. Identified variants were annotated with Variant Effect Predictor (22). TMB (Tumor mutational burden) was calculated as the number of somatic, base substitution, and indel mutations per megabase of exome examined.

RNA-seq analyses for pt#006 and vaccine cell lines were previously described (9, 10). RNASeq data from the pt#006 tumor were uploaded to the European Nucleotide Archive (ENA, EMBL EBI); the corresponding accession number being PRJEB23421, ENA.

To determine/extract neoepitope candidates, MuPeXI 1.1.3 (23) was applied, using as input data the SNVs called by MuTect2, the HLA of the patient and transcript abundance in TPM (transcript per kilobase million) obtained from RNAseq data. To rank the candidates, we also calculated the affinity of *wt* and mutant peptides to the patient's HLAs using NetMHCpan 4.0 (24) and the similarities between *wt* and mutant peptides by applying the alignment-free Kernel Distance. Based on these predictions, three groups of neoepitope candidates were defined. The first group (A) contained candidates in which the mutant peptide has binding rank <2 and *wt* had poor binding to the patient‘s HLA (rank > 5). The second group (B) contained candidates in which both the mutant and *wt* peptides have binding to the patient's HLA (rank <2) and the similarity between mutant and *wt* was low. The third group (negative control) contained candidates in which the mutant peptide showed poor binding to patient's HLA (rank > 5), but a higher binding to *wt* HLA (rank <2). In all groups, peptides were sorted by predicted ranks of mutant binding affinity, *wt* binding affinity, and mutant similarity to *wt* (Supplementary Figure 2).

### Prediction of Neoepitope Binding to HLA Class II Molecules

Binding affinity predictions to the patient's HLA class II molecules were performed using NetMHCIIpan 3.2 (25) for 15-mers contained within neoepitope source proteins with mutation included. We selected promiscuous (binding to at least 2 HLA molecules) and strong binder (rank <2) peptides containing the entire tested neoepitope in the 15-mer and at least 7 amino acids of the neoepitope in the HLA-II binding core.

### IFNγ ELISPOT Assay

PBMC samples were thawed and seeded (1 × 10^6^) in 1 ml of CTL medium consisting of RPMI 1,640 supplemented with 10% heat-inactivated human AB sera, 2 mM glutamine, 100 U/mL penicillin, 100 μg/ml streptomycin, 2.5 mM HEPES, and 50 U/mL IL-2, in 24-well plates. PBMC were stimulated with peptides (10 μg/ml) derived from TAAs or candidate neoAgs, and cultured at 37°C, in 5% CO_2_ for 12 days (effector cells). Every 3 days, fresh CTL medium with IL-2 was added. At day 10, additional PBMC samples were thawed, percentages of CD20^+^ and CD14^+^ cells (Ag presenting cells, APC) were recorded by flow cytometry, and cells were pulsed with peptides during 48 h. At day 12, APC were treated with mitomycin C (50 μg/ml) during 1 h, washed twice with PBS, and resuspended in RPMI 1,640 supplemented with 10% FBS. In addition, effector cells were collected, washed, and resuspended in RPMI 1,640 supplemented with 10% FBS. Effector cells (3–4 x 10^4^) were seeded in 96-well plates (previously coated with 5 μg/ml mouse anti-human IFNγ) and APC were added in a 4:1 ratio, 6.5 × 10^4^ APC/well (1–1.5 × 10^4^ CD20^+^ plus CD14^+^ cells) and cultured O.N. As a positive control, PBMC (3–4 × 10^4^) were seeded and stimulated with 30 ng/ml OKT3 plus 1/1,000 PHA (M form, Gibco Life Technologies). As a negative control, non-stimulated cells were co-cultured with non-pulsed APC. Each experimental condition was performed in triplicate. For each patient, background baseline was calculated as the average number of spots present in non-stimulated cells for each time point sample (PRE-P3).

ELISPOT plates were developed as previously described (12). Plates were scanned using an AID*i*SPOT ELR088IFL analyzer to quantify the number of spots per well; 350 spots/well were set as the maximum quantification limit. An IFNγ-ELISPOT score was calculated as the sum of immune response magnitude (the sum of mean spots/10^5^ PBMC obtained for each positive Ag/number of positive Ags) and diversity (number of positive peptides/total peptides tested).

### CD4 T-Helper Subsets Assessment Throughout Vaccination Protocol

CD4^+^T-cells were purified from PRE, P1, P2, and P3 samples of 13 vaccinated patients (CD4^+^ T Cell Isolation Kit II, human, Miltenyi Biotec). Total RNA (4 × 10^6^ cells) was extracted using TRIzol reagent, and cDNA was generated by reverse transcription with SuperScript II according to manufacturer's instructions (Invitrogen). Primer sequences and extended protocols are available in Supplementary File 2.

### Flow Cytometry

To characterize their phenotype, *in vitro* stimulated Pt#006 PBMC were incubated with anti-human mAbs: APC-H7-CD3 (clone SK7), PerCP-Cy5.5-CD4 (clone RPA-T4), PE-Cy7-CD8 (clone RPA-T8), APC- PD-1 (clone MIH4), APC-CD45RO (clone UCHL1), FITC-HLA-DR (clone G46-6) and PE-CCR7 (clone 150503). Lymphocytes were gated in FSC/SSC dot plot (≥30,000 events), CD4+ and CD8+ T cells were gated within CD3+ cells. Isotype-matched irrelevant mAbs were used as negative controls. All samples were acquired on a BD FACSCanto using FACSDiva software (BD Biosciences) and analyzed with FlowJo 10.0.7 software (USA).

To stain intracellular cytokines, PBMC were thawed and resuspended in RPMI plus 10% FBS supplemented with 50 U/ml IL-2 and stimulated or not with CSF-470 lysate for 10 days, and boosted O.N on day 10. At day 11, cells were centrifuged, resuspended in the same media supplemented with 50 μM PMA, 1 mg/ml ionomycin and Golgi stop BD (0.26 % monensin) and incubated during 3.5 h at 37°C. The cells were then washed, stained with a fixable viability dye FVD eF780 (eBioscience), or Zombie Violet 421 (Biolegend), incubated with Cytofix BD during 15 min at room temperature and washed with PBS. The cells were then permeabilized and blocked with 3% goat serum and stained with PE-Cy7-CD4 (OKT4), PE-IL-10 (JES3-9D7), PE-IL-4 (8D4-8), Alexa Fluor 647-IFNγ (4SB3), purchased from Biolegend. BV421-IL-2 (5344.111) was from BD Biosciences.

### Tumor Cell Lysis Assay

An autologous tumor cell line (Pt#006-T) was established from pt#006, as previously described (9). These cells were used as target cells in an *in vitro* calcein release assay. HLA class I and PMEL/gp100 expression in target cells was verified by FACS (Supplementary File 2 and Supplementary Figure 3). Pt#006-T cells were labeled with 10 μM calcein-acetoxymethyl for 30 min at 37°C, washed twice and resuspended in serum-free assay medium (AIMV, Life Technologies). As effector cells, pt#006 PBMC were stimulated for 12 days as described before, with either CSF-470 vaccine or autologous tumor lysate in a 3:1 ratio (PBMC: Tumor cell lysate) or with peptides with a positive response in the ELISPOT assay. Also, in order to generate an oligoclonal culture of neoantigen-specific effector cells, an *in vitro* expansion protocol was performed before the lysis assay. Briefly, on day 0 pt#006 PBMC (5 × 10^5^) were plated with 5 × 10^6^ 66 Gy gamma-irradiated EBV cells and 25 × 10^6^ 33 Gy gamma-irradiated allogeneic PBLs as feeder layers in T-25 culture flasks and stimulated with OKT3 (30 ng/ml) and with the pool of the 9 positive neoantigen-derived peptides (final concentration 10 μg/ml/peptide) (26). Culture medium was supplemented on day 1 with IL-2 (50 U/ml) and subsequently every 3 days throughout the culture. After 11 days of culture, cells were re-stimulated overnight with the pool of peptides. In all cases, lysis assay was performed on day 12. Target cells were centrifuged, resuspended in assay medium and seeded in 96-well plates (5 × 10^4^ cells/well). Different effector: target ratios were tested in quadruplicate. For spontaneous and maximum release, targets were incubated without effectors in assay medium alone or assay medium plus 1% Triton X-100, respectively. Plates were incubated for 4 h at 37°C in 5% CO_2_, centrifuged, and calcein release was quantified in supernatants in a fluorimeter (485/520 nm OPTIMA, BMG Labtech). The specific lysis (%) was calculated as: (experimental release—spontaneous release)/(maximum release—spontaneous release) ×100.

### Statistical Analysis

Correlation between DTH and INFγ ELISPOT score throughout vaccination was determined by Pearson's coefficient. Differences in T-bet, GATA-3, and Foxp3 mRNA levels between PBMC from high-DMFS and low-DMFS patients were assessed by Mann-Whitney test (*p* = 0.05). IFNγ+/IL-4+ and IFNγ+/IL-10+ ratios differences in PRE and P1 samples from high and low-DMFS patients were analyzed by Wilcoxon test. Statistical significance was set at *p* < 0.05.

## Results

### CSF-470 Vaccination Induced a Progressive Increase in CD8+ and CD4+T Cells Specific for Shared Non-mutated TAAs

**Figure 1:**
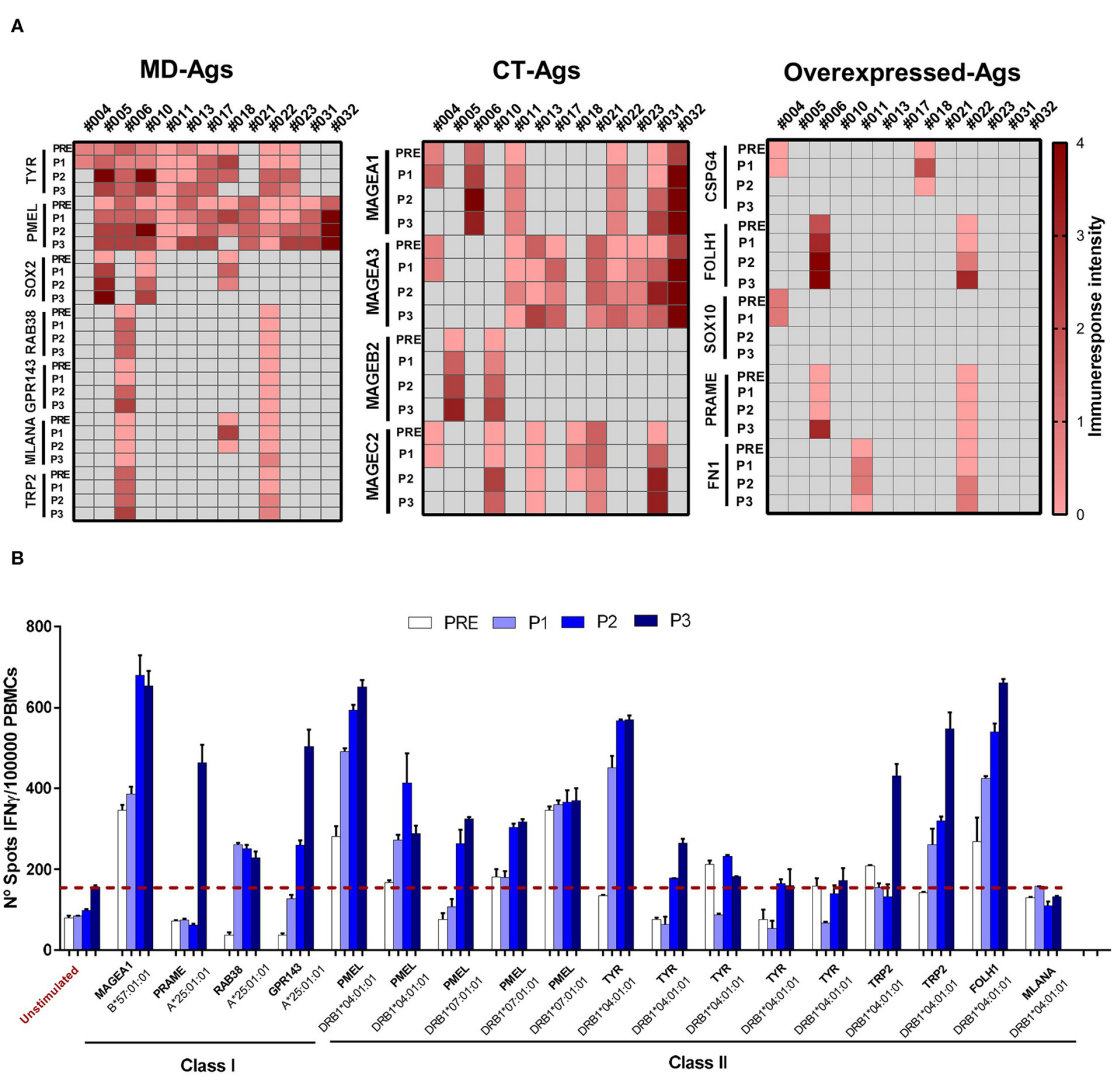
Assessment of T-cell responses against class I and class II TAAs-derived peptides throughout vaccination protocol. PBMC were cultured during 12 days with the corresponding target peptide (effector-cells). At day 13 peptide-loaded mitomycin C-treated APC and effector cells were seeded in ELISPOT plates and cultured O.N as described under Methods. At day 14, cells were removed and IFNγ spots were developed and quantified (up to 350 spots/well). **(A)** T-cell response triggered by TAA-derived peptides in 13 patients treated with CSF-470 vaccine. Each heat map column shows individual patient‘s response against different Ags (rows) of three categories: MD, CT, and overexpressed Ags. Intensity scale reflects the mean number of spots of all evaluated peptides for each Ags/10^5^ effector cells, at the different time points (scale: 0 = no response, 1 = <175 spots, 2 = range 176–350, 3 = range 351–525, 4 = range 526–700). Black cells indicate neither peptide nor sample available for the patient. **(B)** Peptide testing for pt#006: the number of IFNγ spots/10^5^ effector cells at different time points during the vaccination protocol (PRE to P3) are depicted. Peptide names and the corresponding HLA-presenting alleles are shown. Average of non-stimulated effector cells cultured with non-pulsed APC were used as a control to set unspecific spots (red-dashed line). Each sample was tested in triplicate; mean values + SD are shown. APC, antigen presenting cells; ON, overnight; MD, melanoma differentiation antigens; CT, cancer testis antigens.

We previously demonstrated that during CSF-470 treatment T and B cell responses were triggered against the vaccine lysate in all vaccinated patients, and for 2 patients we could verify an immune response against autologous tumor lysates. In contrast, patients treated with intermediate dose IFN-α2b (comparison arm) showed undetectable or lower reactivity which was not modulated by treatment (9, 12). In a subset of 13 out of 19 vaccinated patients that completed the 2-year clinical study (Supplementary File 1, ST1), and from whom we had enough cryopreserved PBMC of each time point (PRE/P1/P2/P3), we analyzed the T cell response to identify the Ags recognized after vaccination. For each patient we stimulated PRE/P1/P2/P3 PBMC *in vitro* with selected HLA class I and class II-restricted peptides (Supplementary File 1, ST2–ST4) from shared, non-mutated TAAs that were expressed in the vaccine as described under Methods (9). IFNγ release was quantified by ELISPOT. In the case of PMEL/gp100 and Tyrosinase Ags, their expression was verified by immunohistochemical staining in tumor biopsies (Supplementary File 1, ST5). As observed in Figure 1A and Supplementary File 3, most patients developed a robust immune response against peptides from TAAs, mostly to PMEL/gp100 and Tyrosinase. This response was low or undetectable in PRE samples and increased progressively in P1–P3 samples. The ELISPOT assay was limited with a quantitative sensitivity of ≤ 350 spots/well, however, we must note that some samples were qualitatively above this threshold. Since patient's PBMC samples collected were scarce, dilution analysis was not possible. For patient #006, RNAseq of the autologous tumor (Pt#006 C-mts) revealed the expression of several TAAs, many of them shared with the CSF-470 vaccine (10). In Figure 1B detailed results are shown for pt#006. Both HLA class I and class II-restricted peptides derived from non-mutated TAAs were able to induce IFNγ release after *in vitro* boost and in most cases this response was higher post-vaccination.

### DTH Reaction to CSF-470 Vaccine Cells Without Adjuvants Correlated With Immune Response Against TAAs

**Figure 2:**
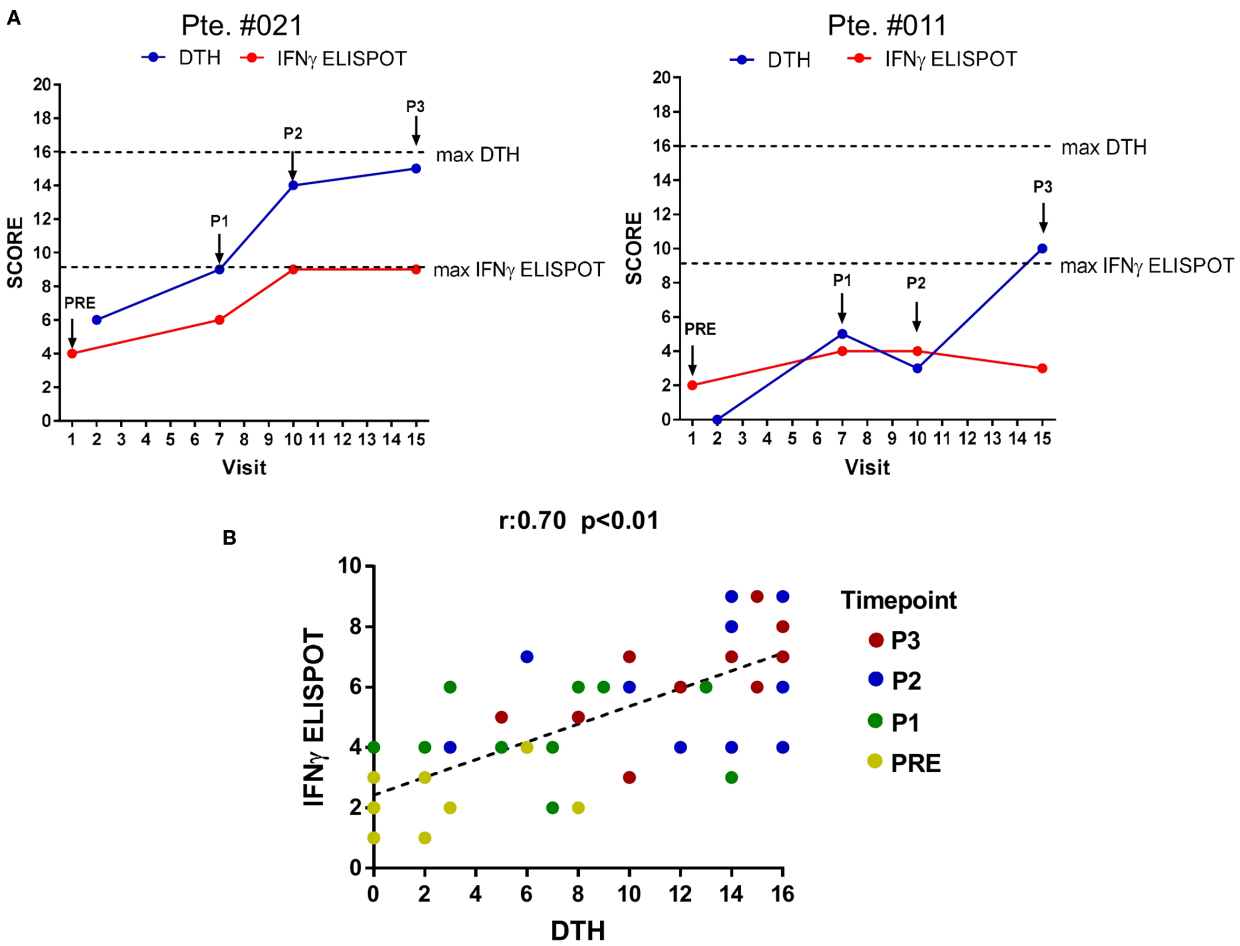
Correlation between DTH and IFNγ-ELISPOT immune response. DTH and IFNγ-ELISPOT score were calculated as described under Methods. **(A)** DTH and IFNγ-ELISPOT score evolution for two representative patients. Dashed-lines represent highest score value (blue line: DTH score, red line: IFNγ-ELISPOT score). Arrows indicate blood samples timepoints. **(B)** Global correlation between DTH-score and IFNγ-ELISPOT score of the 13 patients analyzed. Statistically significant correlation was evaluated by Pearson coefficient (*r* = 0.7; *p* < 0.01). Different time points are displayed with different colored dots. DTH, delayed-type hypersensitivity test.

In the Phase II CASVAC-0401 study, with a mean follow-up >3 yrs, a statistically significant benefit in terms of DMFS was reported for CM patients that received CSF-470 vaccine plus BCG and GM-CSF (13 vaccinations in 2 yr) vs. intermediate dose IFN-α2b-treated pts (*p* = 0.022) (9). An update of patient's status is provided in Supplementary File 1, ST1. DTH response is frequently used as the primary measure of the ability to immunize patients to tumor cells or specific tumor antigens (27, 28). DTH skin test increased in every vaccinated patient after receiving CSF-470 and, after 5 vaccinations, it was higher in patients with no evidence of disease (NED) as compared to those with progressive disease (PRO) (9). We asked whether the development of the DTH response, measured throughout CSF-470 vaccination, correlated to our *in vitro* assessment of peripheral blood TAAs-specific T-cell responses. To that aim, a score that combines the diversity and magnitude of the IFNγ-ELISPOT response against TAAs for 13 evaluable patients was calculated and correlated with DTH score. As observed in Figure 2, a positive correlation was found beetwen DTH and ELISPOT scores (*r* = 0.7; *p* < 0.01).

### GATA-3 and Foxp3 Upregulation in CD4+ Cells 6 Months After Vaccination Start Is Associated With Low DMFS

**Figure 3:**
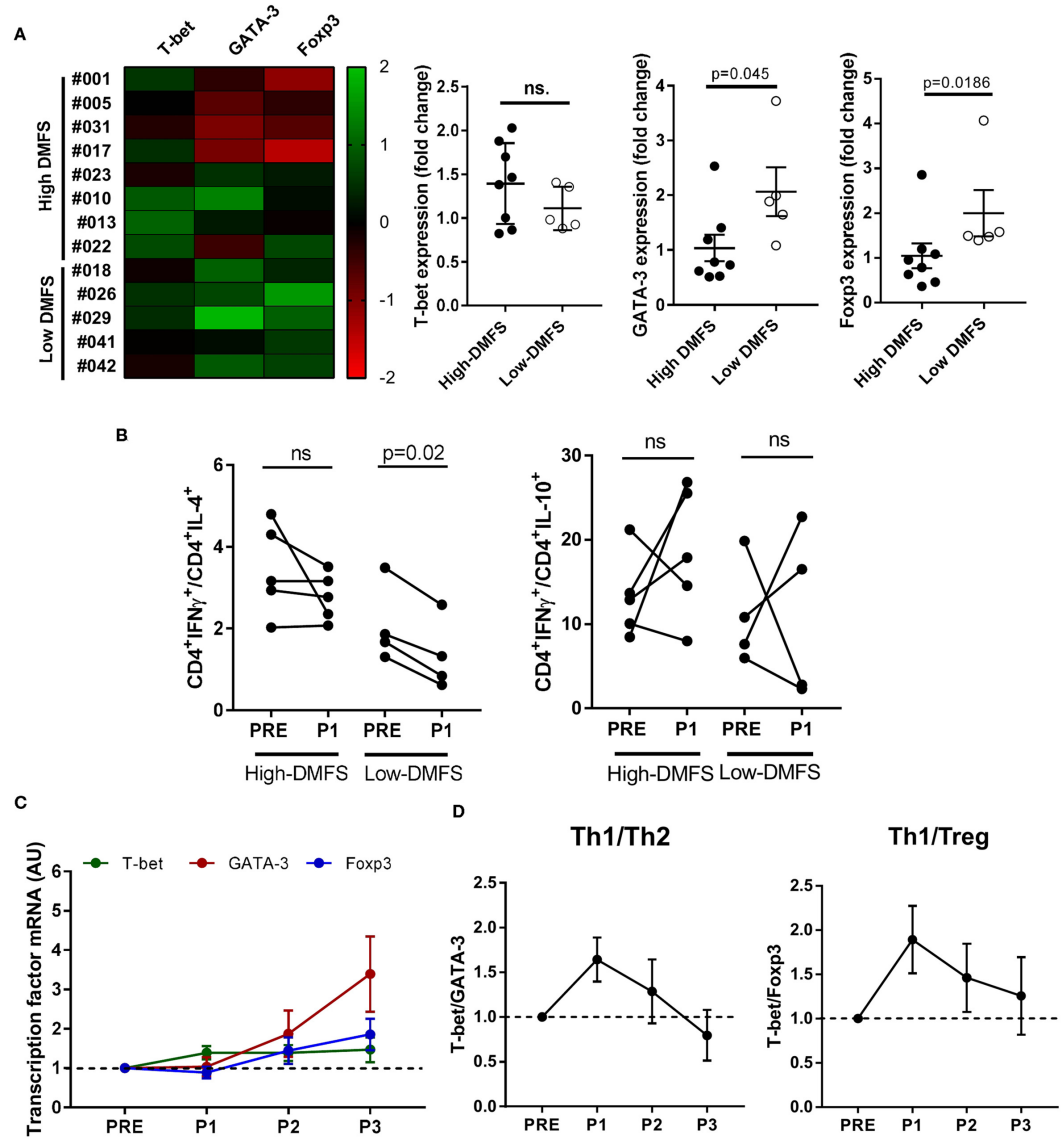
CD4+ T-helper subsets modulation after CSF-470 vaccination. CD4+-cells were isolated from PBMC samples (PRE-P3), and the expression of the main CD4-profile transcription factors T-bet (Th1), GATA-3 (Th2), and Foxp3 (Treg) was determined by qRT-PCR. **(A)** Patients were classified according to DMFS in low (≤ 12 months) (*n* = 5) or high (> 25 months) (*n* = 8). Expression heat map of transcription factors variation (columns) between the PRE and P1 samples for each patient (rows) was build. Differences in expression between low and high-DMFS patients were statistically compared with Mann-Whitney test (*p* < 0.05). **(B)** Intracellular cytokines staining was performed as described under Methods in PRE and P1 samples from high DMFS (*n* =5) and low DMFS (*n* = 4) patients. Depicted are IFNγ+/IL-4+ and IFNγ+/IL-10+ percentage ratios in CD4+ cells between PRE and P1 samples. Statistical differences were evaluated with Wilcoxon test. **(C)** T-helper subsets transcription factors expression in PRE, P1, P2, and P3 samples. **(D)** Th1/Th2 and Th1/Treg ratios throughout vaccination protocol. **(C,D)** include data only from long-term patients (high-DMFS). Dashed line represents relative baseline expression/ratio. DMFS, distant metastasis free survival.

Besides the increase in cytotoxic CD8+ T cells recognizing HLA class I–restricted peptides, many of the recognized peptides were HLA class II-restricted and thus a CD4+T cells response was triggered. Since CD4+T cells act as immune response helpers modulating the fate of such responses depending on they functional phenotype, we evaluated the expression of T-helper subset master transcription factors T-bet (Th1), GATA-3 (Th2), and Foxp3 (Tregs) in CD4+T cells purified from PRE, P1, P2, and P3 PBMC samples with different clinical outcomes (*n* = 13). We classified patients in low (1–12 months) and high DMFS (≥25 months) considering whether they relapsed early or later after vaccination (Figure 3 and Supplementary File 1, ST6). Interestingly, low DMFS patients exhibited a higher expression of GATA-3 and Foxp3 after the first 6 months of vaccination (6 doses) than high-DMFS patients. T-bet expression was slightly higher in the latter group, although the increase was not statistically significant (Figure 3A). In order to confirm transcriptional results, we evaluated the expression of cytokines associated with different subsets: IFNγ (Th1), IL-4 (Th2), and IL-10 (Treg) by intracellular staining of PBMCs from PRE and P1 samples. This analysis was performed for 9 patients, from whom enough PRE and P1 samples were available (high DMFS, *n* = 5; low DMFS, *n* = 4). By analyzing the variation of IFNγ+/IL-4+ and IFNγ+/IL-10+ between PRE and P1 samples we observed that in low-DMFS patients IFNγ +/IL4+ ratio significantly decreased after vaccination, suggesting an increase in Th2 response, whereas no significant changes were observed in high-DMFS patients. IFNγ+/IL-10+ ratio increased after vaccination in both groups of patients; this variation was not statistically significant (Figure 3B). Interestingly, IL-2-producing CD4+ T cells from high-DMFS patients produced more IFNγ in response to *in vitro* stimulation with CSF-470 lysate, both in PRE and P1 samples, as compared to low-DMFS patients. However, no differences were observed for IL-4 and IL-10 production (Supplementary Figure 4). For high-DMFS patients we were able to analyze transcription factors expression evolution from PRE to P3 samples. GATA-3 and Foxp3 start to be upregulated from P2 until the end of the protocol, while the expression of T-bet increases at the beginning of treatment and remains constant (Figure 3C). T-bet/GATA-3 (Th1/Th2) and T-bet/Foxp3 (Th1/Treg) ratios demonstrated that although the initial balance favored a Th1 response at P1 and P2, both ratios decreased at the end of the protocol (P3), suggesting a weakening of the type 1 response (Figure 3D).

### Immune Response Against Predicted neoAgs Increase After CSF-470 Vaccination in Patient#006

Among vaccinated patients, pt#006 developed a cutaneous metastasis (C-mts) at the end of the 2-yr protocol. Access to the fresh tumor biopsy allowed us to perform paired whole exon sequencing to obtain somatic mutations, RNAseq for tumor expression analyses, and the establishment of an autologous melanoma cell line for *in vitro* experiments (10). This circumstance gave us the opportunity to analyze the immune response to tumor neoantigens (neoAgs) and test autologous tumor cell lysis by immune effectors obtained before and after vaccination.

**Figure 4:**
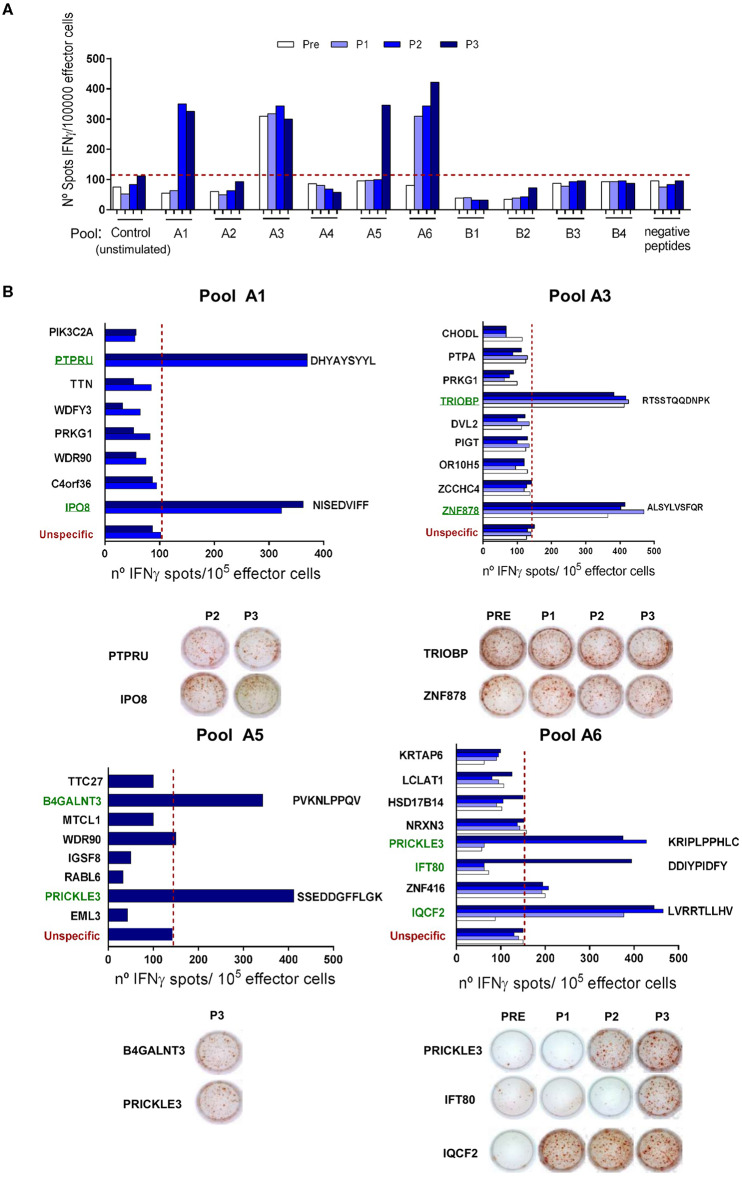
NeoAgs screening and identification. **(A)** At a screening phase, 6 pools of 8–9 peptides from group A (pools A1–A6), 4 pools of 6 peptides from group B (pools B1–B4) and 1 pool of 9 negative peptides were tested following the same protocol described for TAA-derived peptides. Results are shown as IFNγ spots/1 × 10^5^ effector cells. **(B)** Positive pools from the screening phase were opened and the peptides were evaluated individually. Only those time points where a positive response to the peptide pools was seen were evaluated. Peptide sequences for positive neoAgs are displayed next to the bars. Representative ELISPOT pictures are shown for each peptide. For both assays, average of non-stimulated effector cells cultured with non-pulsed APC were used as a control to set unspecific spots (red-dashed line). Each sample was tested in triplicate, shown are the mean values.

Pt#006 C-mts had a TMB = 14.8, with many missense mutations suggesting that immunogenic neoAgs might have been generated (Supplementary File 4, ST1). We designed a pipeline to predict HLA Class I-restricted neoAgs after identification of tumor somatic mutations. Based on self-similarity and predicted binding to pt#006 HLA molecules (Supplementary File 4, ST2) we defined two groups of peptides: (i) those in which the mutant peptide had higher HLA binding potential compared to the wild-type (group A) and (ii) those in which HLA binding was similar for the mutant and wild-type peptides, although both peptides shared low sequence similarity (group B). We evaluated by ELISPOT 49 peptides of group A, and 24 peptides of group B. In an initial screening phase, we assessed 6 pools (A1–A6) of 8–9 peptides each (group A), and 4 pools (B1–B4) of 6 peptides each (group B). We were able to detect IFNγ spots in 4 group A peptide pools (A1, A2, A5, and A6); whereas none of the group B or negative peptide pools induced IFNγ release (Figure 4A). The immunogenicity of each peptide from the positive pools was evaluated separately. We found IFNγ production in response to 9 peptides: two peptides each from groups A1, A3, A5, and 3 from A6 (Figure 4B and Supplementary File 4, ST3). Pre-existing responses against 2/9 immunogenic neoepitopes were found, and for 7/9 neoAgs IFNγ response was found only after vaccination.

**Figure 5:**
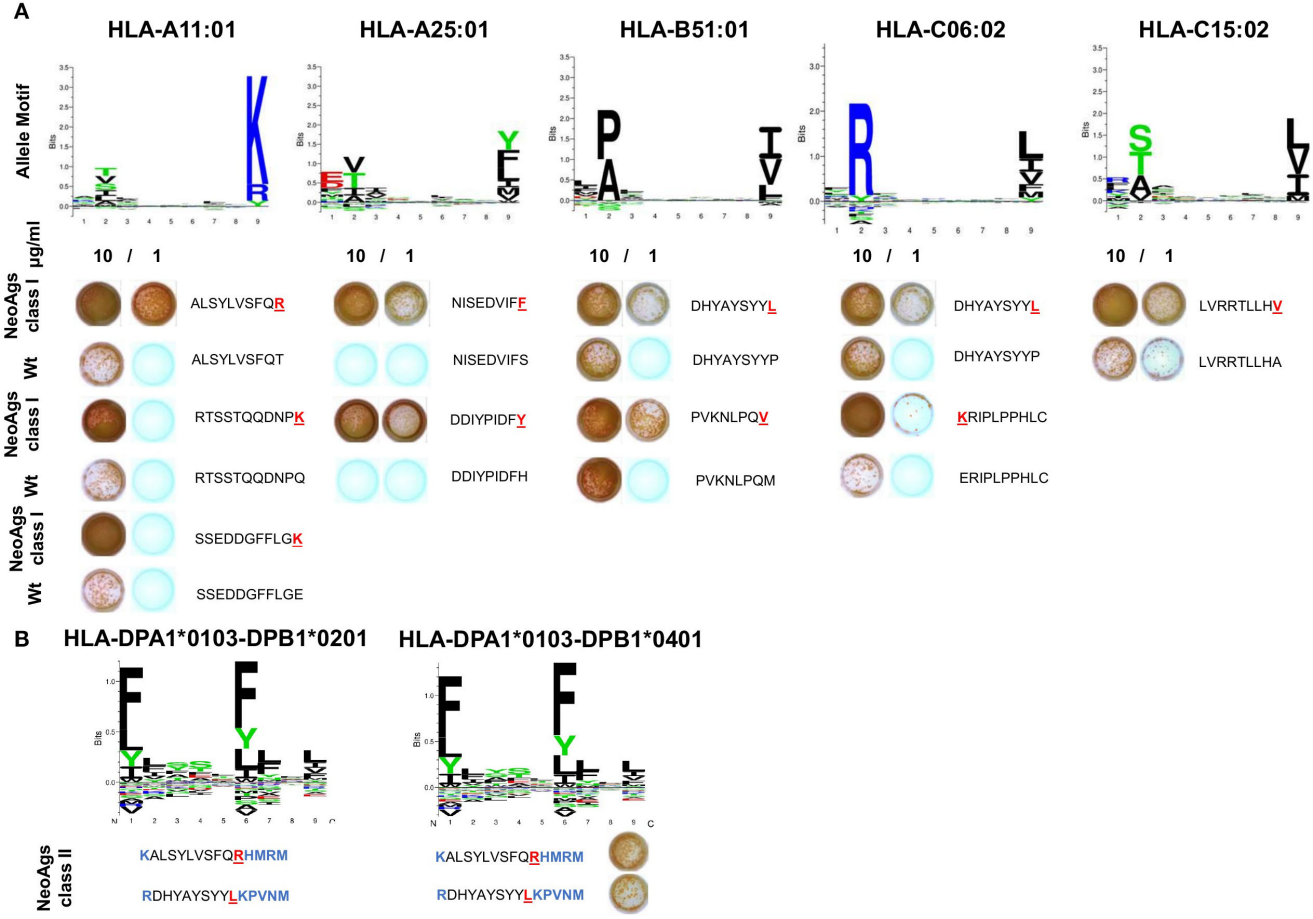
Validation of structural differences and immunological reactivity between neoantigenic and *wt* peptides. **(A)** The binding motifs for Pt#006 HLA class I haplotypes were obtained from NetMHCpan 4.0. PBMC were stimulated as described before with 10 or 1 μg/ml peptides for each of the 9 identified neoAgs (wt vs. mutated) and tested by IFNγ ELISPOT. Peptide sequences are shown, with mutated residues highlighted in red. **(B)** The binding motifs for pt#006 HLA class II haplotypes were obtained from NetMHCIIpan (25). For two neoAgs, HLA class II-restricted peptides containing the point mutation were predicted and tested by ELISPOT as described under Methods (all the residues added are highlighted in blue).

Cross-reactivity to peptides containing the corresponding *wt* sequences of the 9 positive neoAgs was null or very low and only detected at high peptide concentration in the ELISPOT assay. Our results were further supported by the analysis of allele motifs for HLA- molecules of pt#006 that shows that most of the SNVs present in the neoAgs impact in primary or secondary anchor positions and that the mutated residues display higher affinity than wt for HLA molecules. Only DHYAYSYYL (PTPRU) neoAg showed promiscuous binding for two HLA class-I molecules (Figure 5A).

Presence of mutations that gave rise to pt#006 neoAgs as well as amino acid sequences equal to pt#006 neoAgs was verified in the RNASeq data of the four cell lines that compose the CSF-470 vaccine (Supplementary File 2). Only the mutation in IPO8 was detected in the DNA of MEL-XY2 cell line, but with an allele frequency of 0.07 (100% identity blast hits). All remaining blast hits with 100% identity with neoAgs amino acid sequences against vaccine cell lines DNA database correspond to non-coding regions (data not shown).

Finally, for 2/9 HLA class I-restricted immunogenic mutations above described, we synthesized 14-mer peptides predicted to bind to pt#006's HLA class II molecules for ELISPOT testing. IFNγ spots revealed that the same mutations can give rise to HLA class I and class II-restricted neoepitopes, suggesting that CD8+ and CD4+T cell responses targeting pt#006 mutations may occur (Figure 5B).

### PBMC Obtained Post-vaccination With Allogeneic CSF-470 Vaccine Can Lyse Autologous Tumor Cells

Since the frequency of T cells recognizing TAAs and neoAgs, detected by IFNγ production, increased during vaccine stimulation, a relevant question was whether such lymphocytes were able to lyse autologous tumor cells. For that purpose, we performed an *in vitro* lysis assay using Pt#006-T cells as targets of pt#006 PRE, P1, P2, and P3 PBMC samples boosted *in vitro* either with the vaccine cells lysate, autologous tumor cells lysate or peptides that stimulated IFNγ production in the ELISPOT assays.

**Figure 6:**
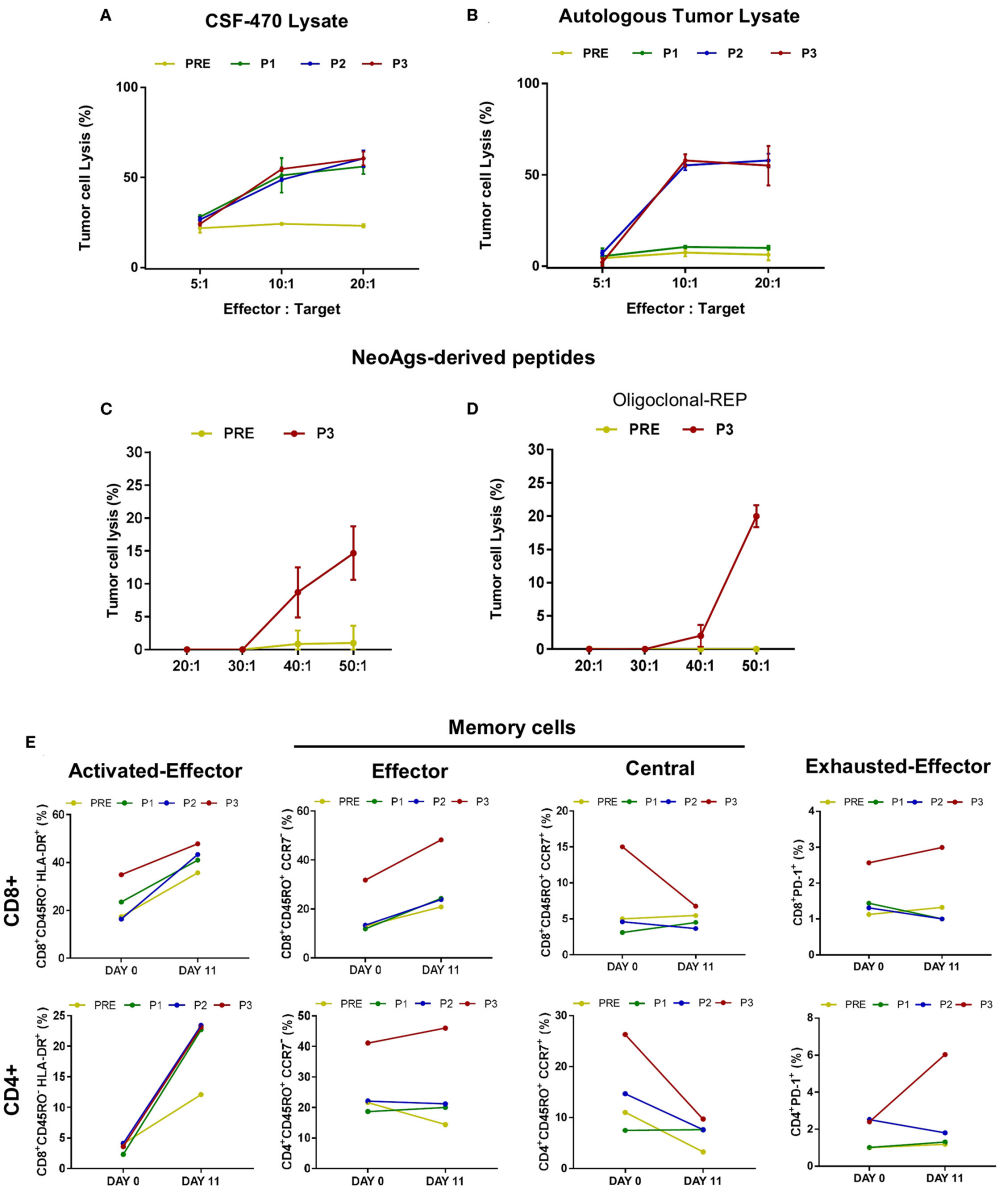
Cytotoxic response triggered by CSF-470 vaccination in pt#006 PBMC samples. Pt#006-T cells lysis was evaluated by the *in vitro* calcein release assay as described under Methods. As effector cells, autologous PBMC were stimulated during 12 days with CSF-470 vaccine lysate **(A)** autologous tumor lysate **(B)**, both in a 3:1 ratio (PBMC: tumor cell lysate), a pool of the 9 identified neoAgs peptides **(C)** or the same pool of 9 neoAgs plus OKT3 and a feeder layer, as described under Methods **(D)**; every peptide was tested at a 10 μg/ml concentration. 5 × 10^4^ target cells/well were seeded in 96-well plates. Different effector: target ratios were tested in quadruplicate. **(E)** T cells stimulated by the autologous tumor cell lysate were analyzed by flow cytometry at days 0 and 11 after stimulation and prior to the Pt#006-T cells lysis assay, to identify CD4+ and CD8+ T cell's phenotype: activated effector cells (CD45RO^−^HLA DR+), memory subsets: effector memory (CD45RO^+^CCR7–); central memory (CD45RO+ CCR7+) cells and effector exhausted (PD-1+) cells in the different PBMC samples (PRE-P3).

Interestingly, as observed in Figures 6A,B, post-vaccination PBMC were able to lyse ≥50% autologous tumor cells in a 10:1 effector: target ratio when they were boosted *in vitro* with CSF-470 vaccine lysate, probably reflecting stimulation of cytotoxic T cells targeting common/shared TAAs. Similarly, post-vaccination PBMC boosted *in vitro* with autologous tumor cell lysate (including also stimulation of neoAg-reactive T cells) lysed ~ 50% of target cells (P2 and P3). Less efficient, although, was tumor cell lysis after stimulation with peptides (Figures 6C,D). Stimulation with the pool of 9 HLA class I-restricted neoAgs boosted P3 lymphocytes to produce ~15% Pt#006-T cells lysis, only at a 50:1 effector: target ratio. *In vitro* expansion of P3 lymphocytes in the presence of a feeder layer, OKT3 stimulation and the pool of 9 neoAgs achieved lysis of 20% pt#006-T cells. In addition, stimulation with class-I peptide derived from PRAME did not induce lysis (not shown).

Due to the scarce PBMC availability, we analyzed the T cell phenotype only of PBMC stimulated *in vitro* with autologous tumor cell lysate prior to the cell lysis assay. Both CD4+ and CD8+ activated effector T cells increased during *in vitro* stimulation, with a higher response for post-vaccination samples (Figure 6E). Of note, CD8+ and CD4+ T cells in P3 sample (after 13 vaccinations) responded with a higher increase in effector/activated cells, also showing more PD1+ cells from the start of culture. Particularly the CD8+ T cells achieved a higher increase in PD1 expression as compared to PRE, P1, and P2 samples, suggesting their Ag-experienced phenotype. On the opposite, central memory T cells (CD4+ and CD8+) decreased but effector memory T cells increased for CD8+ compartment, remaining at constant levels for CD4+ T-cells.

## Discussion

The CSF-470 vaccine offers to the patient's APC a complex array of TAAs from the four melanoma cell lines that form it (9). Some of these Ags will be processed into peptides suitably presented in each patient's HLA-class I and II haplotypes. Many of the peptides that derive from non-mutated genes are highly immunogenic, due to incomplete tolerance to TAAs (29). Our results on 13 vaccinated patients demonstrate that CSF-470 vaccine induces a robust immune response to a wide range of TAAs presented in the context of HLA class I and II molecules. Traditionally, CD8+T cells are recognized as the main antitumor effector cells, since they are endowed with cytotoxic activity, and down-regulation of HLA-class I expression allows tumors to escape elimination (30). Targeting of CD8+T cells to TAAs leading to melanocytes destruction (evidenced as vitiligo) has been reported as an indicator of antitumor immune responses in CM patients that received various types of immunotherapies (31, 32). TAAs like PMEL/gp100, tyrosinase, and several CT Ags have been identified as melanoma regression Ags (33, 34). The roles of non-mutated TAAs were recently reinvigorated as key players in the endogenous anti-melanoma immunity (35, 36) since they may be involved in either CM surveillance or dormancy. In our study, vaccination with the allogeneic CSF-470 vaccine has provided sustained targeting of TAAs. We have shown that most CSF-470 vaccinated patients increased their T cell IFNγ response after recognition of several vaccine-expressed TAA-derived HLA class I and II-restricted peptides. These results raise the question about the possible role of CD4+ T cells expanded after CSF-470 vaccination in targeting and elimination of tumor cells.

Knowledge about CD4+ T lymphocytes role in tumor immunotherapy is limited and mainly unexplored, and may include enhancement of cytotoxic T lymphocyte responses, licensing of APC, or direct cytotoxic function (37). In different experimental models, it was shown that an exclusive tumor Ag-specific CD8+ T cell response, in the absence of an antitumoral CD4+ T cell response, cannot establish long-lasting memory (38, 39). In addition, the elimination of tumor-specific CD4+ T cells compromises secondary antitumor responses after successful primary immunotherapy (40), reinforcing the idea of a necessary CD4+ T cell help. Also, the adoptive transfer of Ag-specific CD4+ T-cells in advanced CM patients can mediate durable complete tumor regressions (41). As shown, transcriptional profiles of CD4+ T cells in CSF-470 vaccinated patients that remained distant metastasis-free were preferentially polarized to a Th1 state. This could be probably related to the use of BCG as an adjuvant for CSF-470 vaccine, since BCG has been demonstrated as a potent inducer of Th1 polarization, i.e., in non-muscle invading bladder cancer patients treated with intravesical BCG (42). Under our context, vaccine-stimulated Ag-specific CD4+T cells may provide help to cytotoxic CD8+T cells and innate immunity cells through release of pro-inflammatory cytokines (as suggested by intracellular cytokine staining in P1 vs. PRE samples); besides, these cells may also contribute to the elimination of residual CM cells (43).

Only a fraction of predicted high affinity HLA class I binding mutant peptides are expected to be naturally presented and immunogenic HLA class I ligands (44). Accordingly, when analyzing pt#006 neoAgs prediction from missense mutations, we observed CD8+ T cell responses against 9/49 (18%) predicted HLA class-I group A peptides. In contrast, none of group B peptides elicited an immune response, suggesting that the relatively simple model of self-similarity applied here is inadequate, and that more refined approaches are needed to identify neoAgs sufficiently dissimilar to HLA bound self-peptides that can be detected by the TCR. Also, two peptides containing neoepitope-generated somatic mutations were predicted to bind to pt#006's HLA class-II alleles, and their immunogenicity was confirmed by IFNγ ELISPOT. Thus, single mutations may give rise to peptides either recognized concomitantly by CD4+ and CD8+ T cells or presented on different HLA class I restriction elements to different CD8+T cells, as previously reported (45).

In the case of pt#006, we previously reported the analysis of the CDR3-T-cell receptor β (TCRβ) repertoire detected in blood throughout immunization, revealing an expansion of selected preexisting and newly arising T cell clones with reduction of others. In a C-mts heavily infiltrated by CD8+ T cells, prevalent clones (~50%) were both new and preexisting, which expanded in blood following CSF-470 immunization. Of note, 51% of the clones present in the C-mts could still be detected in blood by the end of the protocol (10). Therefore, our results strongly suggest that vaccination can induce a persistent antitumor T cell response that can reach tumor sites and recognize TAAs as well as neoAgs.

The immune response to neoAgs either increased or emerged after vaccination with the allogenic CSF-470 vaccine, not expressing pt#006 private neoAgs. Our results support the hypothesis that repeated CSF-470 plus BCG, and GM-CSF vaccination may have caused waves of tumor Ags release from the irradiated vaccine cells, and subsequently, Ag-stimulated CD8+ and CD4+ T cells of the host may have lysed micro-disseminated tumor cells, leading to repetitive endogenous re-stimulation events broadening the immune response. Adjuvant BCG may have fueled such antitumor immunity skewing CD4+ T cells toward a Th1 profile, resulting in a cytokine balance that favored antitumor responses. *In vitro*, we observed a strong and similar cytotoxic activity when the whole vaccine lysate or the autologous tumor lysate were furnished to patient's APC, challenging post-vaccination PBMC with a variety of TAAs (those already tested as well as others unknown) in the first case, and also including patient's neoAgs in the second case. However, a weaker tumor cell lysis was verified after stimulation with the 9 identified neoAgs. This result demonstrates that CSF-470 vaccination induces cytotoxic lymphocytes targeting both TAAs and neoAgs, albeit killing efficiencies toward the different TCR-peptides may vary. Also, the lower abundancy of neoAg-derived peptides in the surface of target cells, as compared to those derived from highly expressed TAAs may account for the lower tumor cell lysis observed.

CSF-470 vaccine plus BCG and GM-CSF produced a clinical response in terms of prolonged DMFS in high-risk CM patients (9). A limitation of our study is the small number of cases. However, we demonstrated here for the first time that vaccination increased the frequency of specific antitumor effectors in a Th1 skewed immune context providing the basis to further explore CSF-470 vaccine immunization.

It would then be possible to potentiate ICKB with previous CSF-470 vaccination since the number and variety of effectors would increase. This hypothesis should be explored in future clinical studies.

## Conclusions

Adjuvant immunotherapy of CM patients with CSF-470 vaccine plus BCG and GM-CSF has shown a significant benefit in DMFS as compared to IFN-α2b in a phase II study. After analysis of the immune responses elicited after the 2-yr vaccination, we conclude that all patients developed a robust CD8+ and CD4+ Th1 cellular response targeting non-mutated shared TAAs. In one patient, we could also verify recognition of neoAgs and autologous tumor cell lysis. Thus, vaccination may have caused waves of tumor Ag release from the irradiated vaccine cells and from the host (autologous) micro-disseminated melanoma cells, leading to repetitive *in vivo* vaccination events that allowed broadening of the immune response toward diverse TAAs and neoAgs. In most vaccinated patients, this immune response may have contributed to local or circulating tumor cell clearance, preventing or delaying melanoma relapse.

## Data Availability Statement

RNASeq data from the pt#006 tumor were uploaded to the European Nucleotide Archive (ENA, EMBL, EBI); the corresponding accession number being PRJEB23421, ENA). WES data from the four human melanoma cell lines that compose CSF-470 vaccine are not publicly available since they are protected by an US patent (12/450,721/US2010183683).

## Ethics Statement

The CASVAC-0401 study was carried out after approval of the Ethics Committee of the Instituto Alexander Fleming, with written informed consent from all participating subjects in agreement with the Declaration of Helsinki. The informed Consent included the authorization to publish the results obtained, providing anonymity was assured. The Ethics Committee of the Instituto Alexander Fleming (Buenos Aires, Argentina) is reputed by the Central Ethics Committee of the City of Buenos Aires (Argentina). The study was also approved by the Argentine Regulatory Agency (ANMAT) (Disposition 1299/09). The patients/participants provided their written informed consent to participate in this study.

## Author Contributions

MB and JM: conception, design, data analysis, interpretation, manuscript writing and they are the Sub and Principal Investigator of the CASVAC-0401 study, respectively. EP: collection and assembly of data, data analysis, and manuscript writing. IC and MN: assembly of data, data analysis, and manuscript writing. MA, AB, PB, and JO: collection and assembly of data. EE, DK, PY, and MN: data analysis and interpretation.

## Conflict of Interest

EE was employed by company T-cure Bioscience Inc. The remaining authors declare that the research was conducted in the absence of any commercial or financial relationships that could be construed as a potential conflict of interest.
