# Supplementary figures and images for: Evaluation of T-Cell Responses Against Shared Melanoma Associated Antigens and Predicted Neoantigens in Cutaneous Melanoma Patients Treated With the CSF-470 Allogeneic Cell Vaccine Plus BCG and GM-CSF
Source: Front Immunol. 2020 Jun 5;11:1147. doi: 10.3389/fimmu.2020.01147 (PMC7290006; doi:10.3389/fimmu.2020.01147)

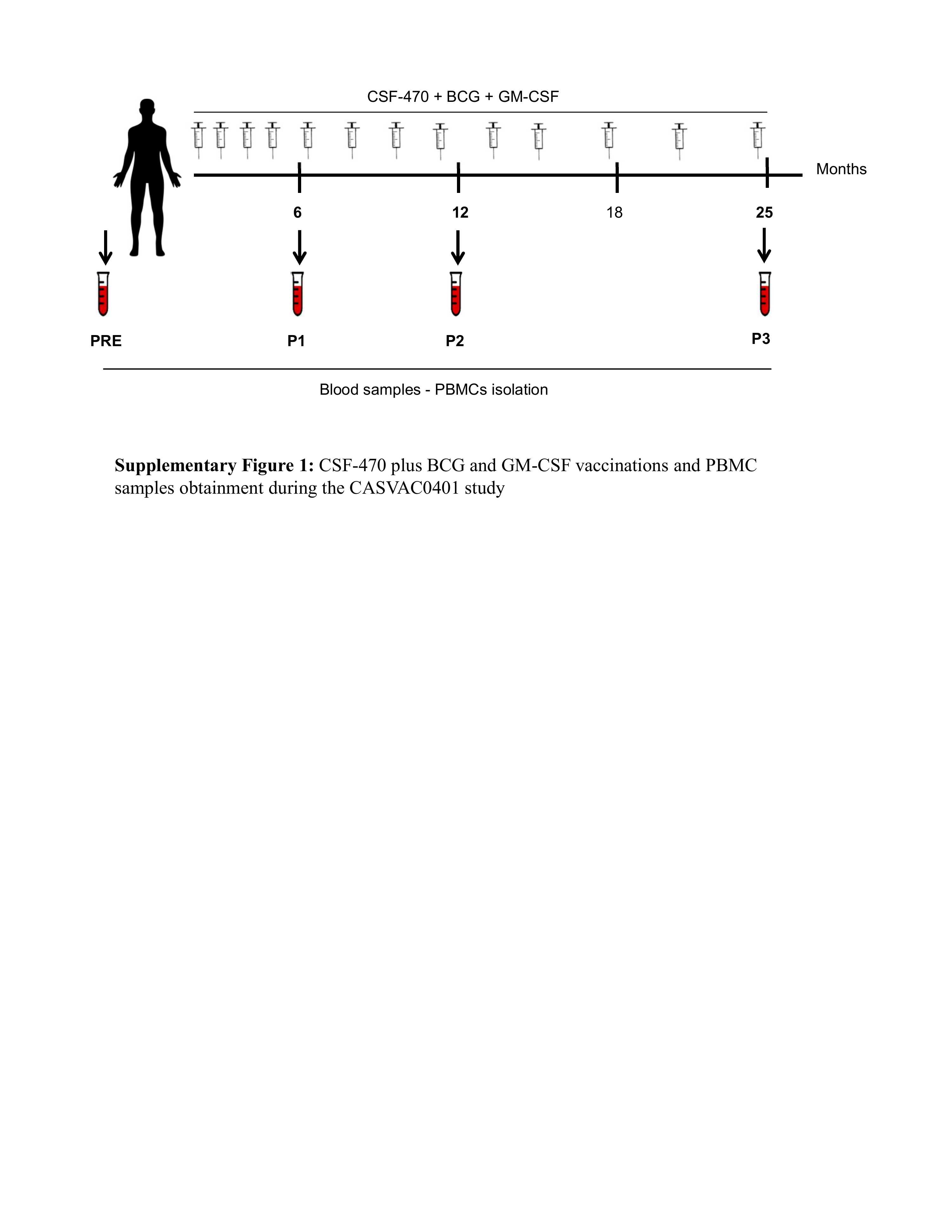

Supplement: Supplementary file 1 [file Image_1.JPEG]

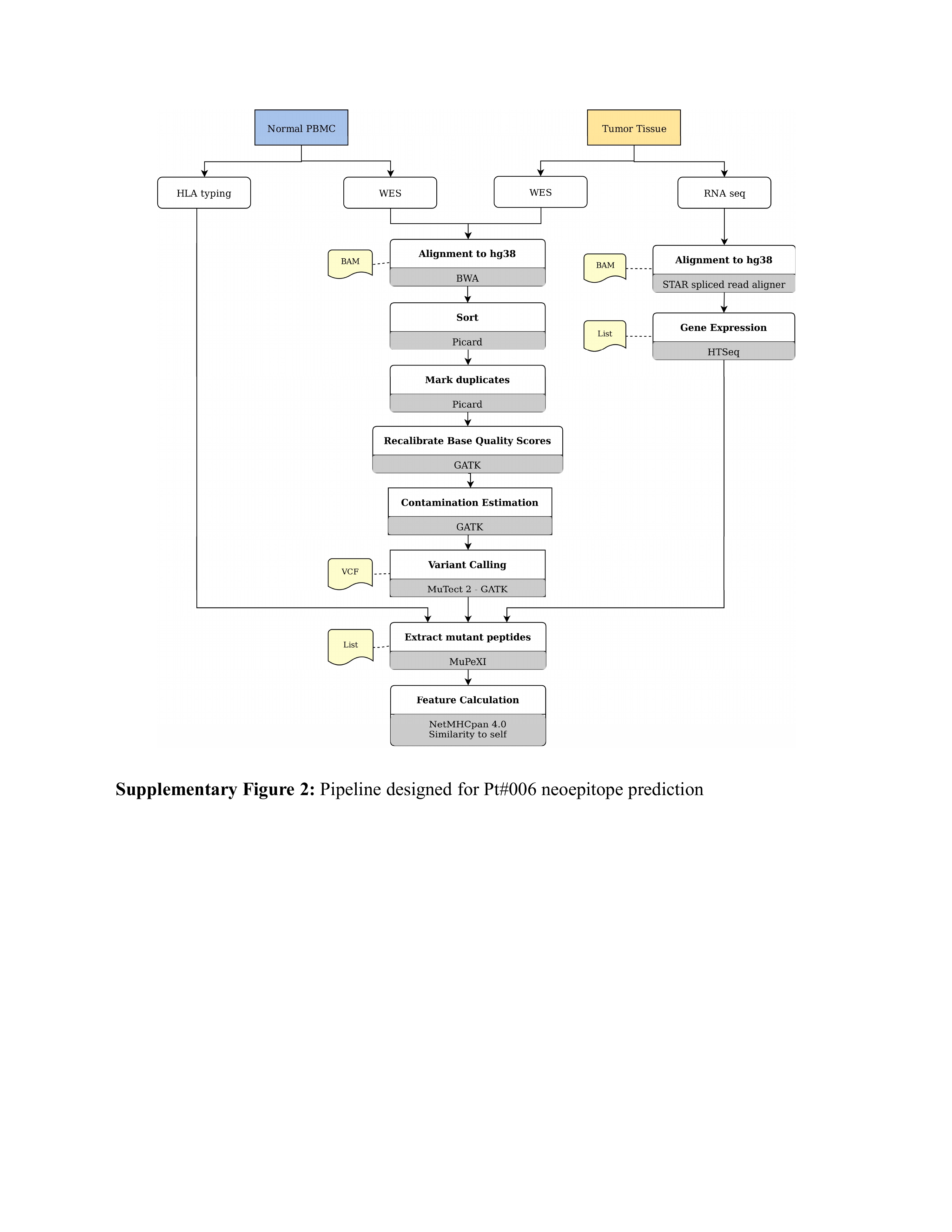

Supplement: Supplementary file 2 [file Image_2.JPEG]

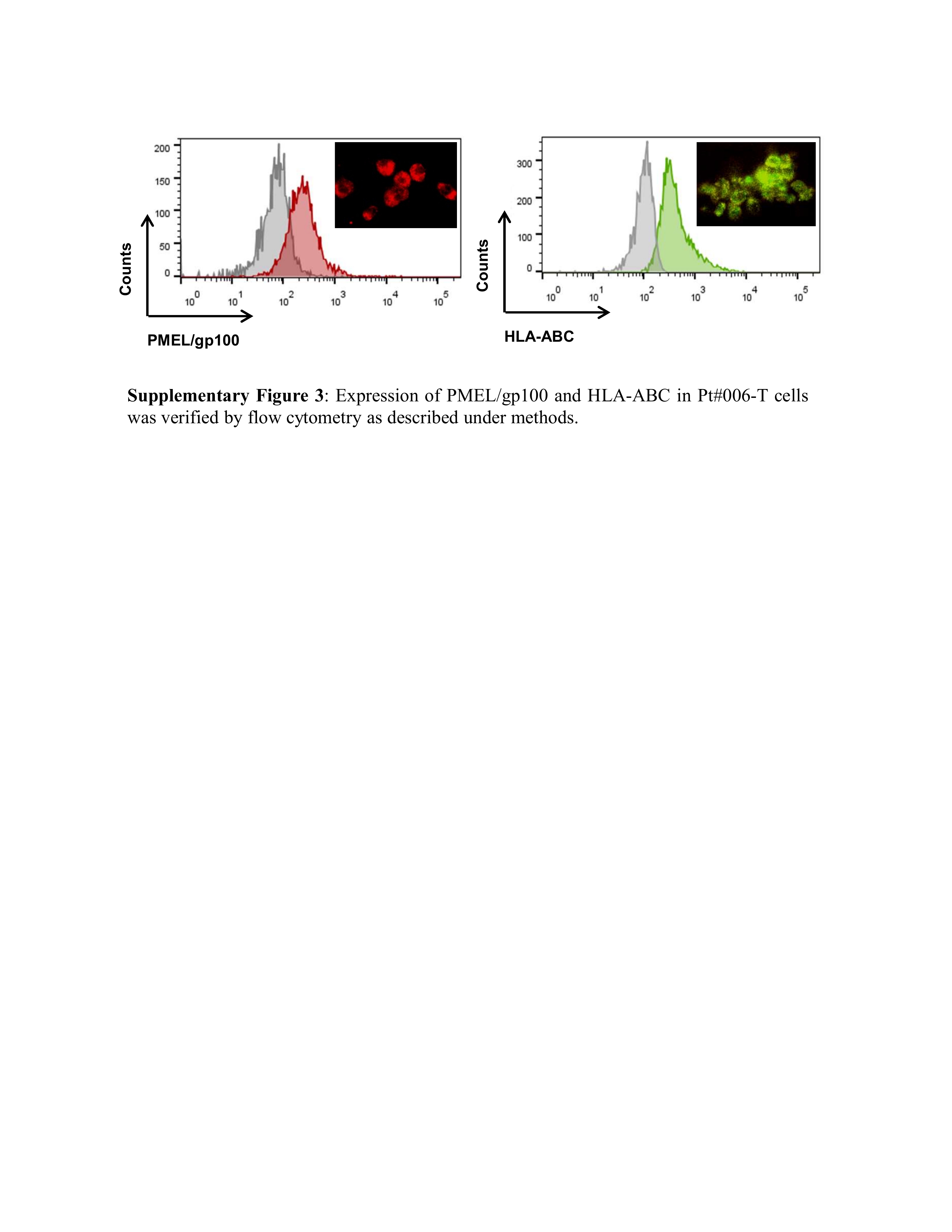

Supplement: Supplementary file 3 [file Image_3.JPEG]

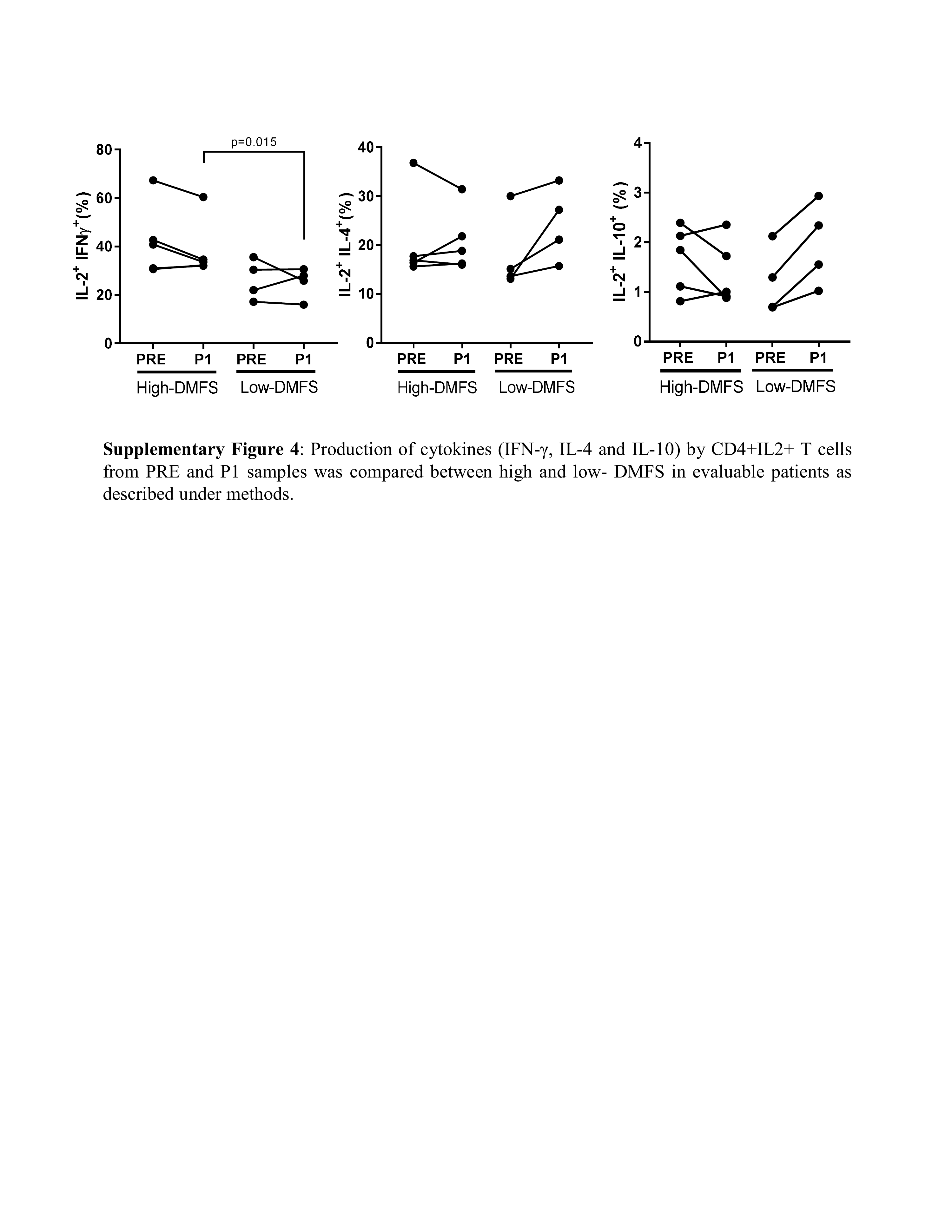

Supplement: Supplementary file 4 [file Image_4.JPEG]
