# Supplementary material for: Evaluation of T-Cell Responses Against Shared Melanoma Associated Antigens and Predicted Neoantigens in Cutaneous Melanoma Patients Treated With the CSF-470 Allogeneic Cell Vaccine Plus BCG and GM-CSF
Source: Front Immunol. 2020 Jun 5;11:1147. doi: 10.3389/fimmu.2020.01147 (PMC7290006; doi:10.3389/fimmu.2020.01147)
Supplement: Supplementary file 5 [file Data_Sheet_1.zip › Supplementary file 2.docx]

Additional file 2. Extended methods

**TAAs expression in tumor biopsies**

Formalin-fixed, paraffin-embedded tumor biopsies from all vaccinated patients were analyzed by immunohistochemistry as previously described (Mordoh, 2017). Monoclonal antibodies used were: PMEL/gp100 (clone HMB45, Dako) and Tyrosinase (clone M3623, Dako).
**CD4 T-helper subsets assessment throughout vaccination protocol.**

Master CD4+ helper subset transcription factors expression was address by qRT-PCR using PowerUp SYBR Green PCR Master Mix in 20 µl reactions. The following primers were purchased from Thermo-Fisher Scientifics:

T-bet Fw 5´-CAGAATGCCGAGATTACTCAG-3´, Rv 5´-GGTTGGGTAGGAGAGGAGAG-3´,

GATA3 Fw 5´-TCCGAGCACAACCACCTTAG 3´, Rv 5´GCCGTTGAGGGTTTCAGAGA3´,

Foxp3 Fw 5’-GCTTCATCTGTGGCATCATC-3’, Rv 5’-TGGAGGAACTCTGGGAATGT-3’.

EEF1A1 Fw 5´-TCGGGCAAGTCCACCACTAC -3´, Rv 5´-CCAAGACCCAGGCATACTTGA-3´,

and used at a final concentration of 250 nM. Reactions were carried out in a QIAGEN Rotor-gene Q Real-Time PCR System. The cycling program used was 50 ˚C for 2 minutes, 95˚C for 1 minute, followed by 40 cycles of 95˚C for 15 seconds, 60˚C for 60 seconds. Data were analyzed using EEF1A1 as a reference gene. Additionally, changes in gene expression were relativized to the PRE sample for each patient.

**Screening of neoepitopes in CSF-470 vaccine cells**

Mutations giving rise to neoepitopes identified for Pt#006 were screened in the melanoma cell lines that compose the vaccine by using IGV. WES and RNAseq samples were aligned to the human reference genome (GRCh38) using Burrows-Wheeler Aligner (BWA) and STAR (v2.5.3a) respectively. Neoepitope aminoacidic sequences were screened in WES and RNAseq data from the vaccine melanoma cell lines by using the BLAST+ (v2.8.1+) tBLASTn tool. DNA and RNA databases for each cell line were constructed with makeblastdb using WES fastq files. BLOSUM62 was used as scoring matrix, expectation value (E) threshold for saving hits was set to 200,000 to retrieve all possible blast hits, threshold for the word to be added to the BLAST lookup table was set to 0.001 and word size was set to 2.

**Flow cytometry**

Pt#006-T cells were incubated with the following fluorochrome-conjugated monoclonal antibodies (mAbs): PMEL/gp100 (Primary mAb, clone HMB45, secondary mAb: Polyclonal goat anti-mouse Ig-PE) and HLA-ABC (clone g46-2.6, FITC) and analyzed by Flow cytometry.
